# Outcomes of critically ill pediatric patients after haploidentical stem cell transplantation

**DOI:** 10.3389/fped.2026.1782412

**Published:** 2026-05-29

**Authors:** Da Hyun Kim, Ho Joon Im, Won Kyoung Jhang, Sung Han Kang

**Affiliations:** 1 Department of Pediatrics, Asan Medical Center Children’s Hospital, University of Ulsan College of Medicine, Seoul, Republic of Korea; 2 Division of Pediatric Hematology–Oncology, Department of Pediatrics, Asan Medical Center Children’s Hospital, University of Ulsan College of Medicine, Seoul, Republic of Korea; 3 Division of Pediatric Critical Care Medicine, Department of Pediatrics, Asan Medical Center Children’s Hospital, University of Ulsan College of Medicine, Seoul, Republic of Korea

**Keywords:** haploidentical peripheral blood hematopoietic stem cell transplantation, ICU, mortality, pediatrics, risk factor

## Abstract

**Background:**

Haploidentical peripheral blood hematopoietic stem cell transplantation (haploPBSCT) is increasingly performed for both malignant and nonmalignant diseases. This study evaluated the outcomes of critically ill patients after haploPBSCT who were admitted to the pediatric intensive care unit (PICU).

**Methods:**

This single-center, retrospective study included pediatric patients who underwent haploPBSCT (*ex vivo* αβ+ T-cell and CD19+ B-cell depleted after 2012) and were admitted to the PICU between 2010 and 2023. Data were collected from electronic medical records. The primary outcomes were 30-day mortality from the time of PICU admission and in-hospital mortality. Secondary analyses examined risk factors associated with these outcomes.

**Results:**

A total of 63 patients met the inclusion criteria. The 30-day and in-hospital mortality rates were 33.3% and 50.8%, respectively. The most common reason for PICU admission was respiratory complications. In multivariate logistic regression analysis, chronic graft-vs.-host-disease was independently associated with 30-day mortality [*p* = 0.043, odds ratio (OR) 4.515; 95% confidence interval (CI): 1.050–19.414], while ventilator use was an independent predictor of in-hospital mortality [*p* = 0.046, OR 5.15 (95% CI: 1.029–25.724)]. Among patients who survived beyond 30 days in the PICU, 11 subsequently died, 10 of whom had received ventilator support for respiratory failure; pneumonia was the most common cause of death.

**Conclusion:**

Thirty-day and in-hospital mortality rates after haploPBSCT were comparable to those reported for allogenic PBSCT. Most critically ill pediatric patients required PICU admission for respiratory complications. Ventilator use independently predicted in-hospital mortality, underscoring the importance of early detection and prevention of respiratory failure following haploPBSCT.

## Introduction

Haploidentical peripheral blood hematopoietic stem cell transplantation (haploPBSCT) is a type of hematopoietic stem cell transplantation in which the donor shares one haplotype, matching half of the human leukocyte antigen (HLA) class I and II with the recipient (1). This approach substantially expands donor availability, enabling transplantation in patients who lack matched sibling or unrelated donors. HaploPBSCT is now widely performed for both malignant and nonmalignant hematologic disorders (2, 3). In recent years, favorable outcomes have been reported in patients with very severe or aplastic anemia and in those with refractory hematologic diseases who underwent haploPBSCT (4–6). Consequently, While allogeneic bone marrow transplantation (BMT) remains the standard of care at the majority of transplant centers worldwide, practice patterns vary considerably across institutions and regions (6, 7). At many centers in South Korea, including our own, allogeneic peripheral blood stem cell transplantation (PBSCT) has become the established standard of care for pediatric patients due to less invasive for donors and less labor-intensive for doctors (8), reflecting both regional practice evolution and accumulated institutional experience. Against this backdrop, the use of haploidentical PBSCT (haploPBSCT) has increased markedly in recent years as an alternative for patients who lack suitable matched donors. Some studies have even suggested that haploPBSCT may offer superior overall survival compared with matched sibling donor alloPBSCT (9). Encouraging results have also been documented in pediatric populations with conditions such as aplastic anemia, leukemia, and sarcoma (2, 10–12).

As the use of haploPBSCT expands, it is important to recognize its potential complications, particularly in patients who become critically ill after transplantation. Outcomes and risk factors for critically ill patients following alloPBSCT have been extensively studied (13–15). A recent large cohort study reported that alloPBSCT recipients requiring ICU management, particularly those who required invasive mechanical ventilator (MV), continued to have poor prognoses (15). Given that haploPBSCT and alloPBSCT differ in conditioning regimens and GVHD prophylactic strategies, the characteristics, severity, and risk factors of post-transplant critical illnesses may also differ between these two transplant types.

**Figure 1:**
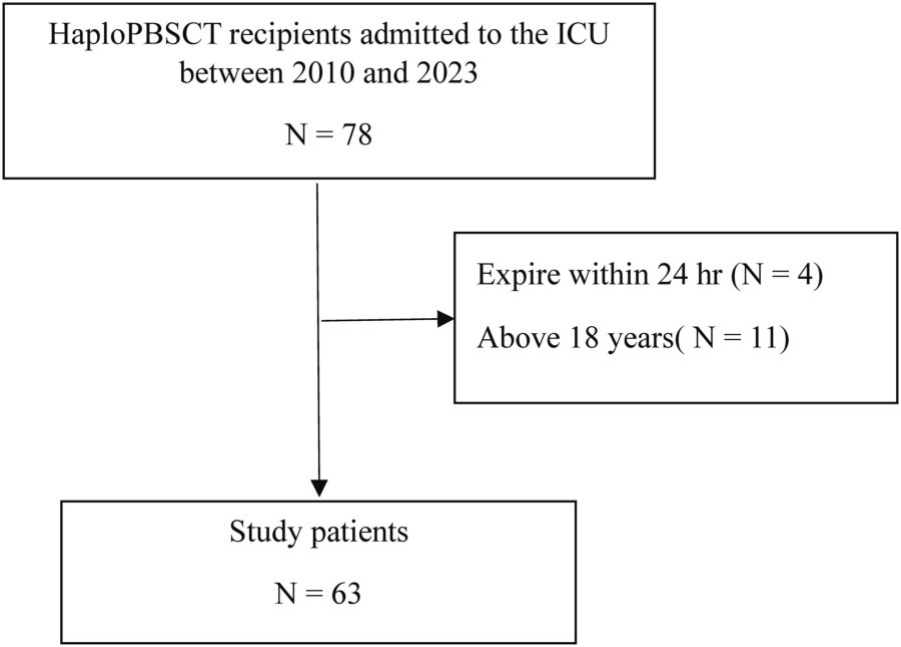
Flow chart.

However, the outcomes and risk profiles of critically ill patients after haploHSCT, particularly in children, remain poorly defined. At our institution, most of haploidentical transplants were performed using *ex vivo* αβ+ T-cell and CD19+ B-cell depletion, a graft manipulation strategy that differs from post-transplant cyclophosphamide-based approaches in terms of immune reconstitution and infection risk. Therefore, this study aimed to investigate the clinical outcomes and associated risk factors in critically ill pediatric patients who underwent haploPBSCT (Figure 1) .

## Methods

### Study population

This single-center, retrospective, observational study reviewed the medical records of all pediatric patients who underwent haploHSCT and were subsequently admitted to the PICU of a tertiary academic referral hospital between January 2010 and December 2023. Patients aged ≥18 years or those who died within 24 h of PICU admission were excluded.

The study was approved by the Institutional Review Board of the University of Ulsan College of Medicine (#2025-0570), and the requirement for informed consent was waived due to the retrospective nature of the study. All procedures were conducted in accordance with the principles of the Declaration of Helsinki.

### Haploidentical hematopoietic cell transplantation procedures

Patients who underwent haploHSCT from 2012 onwards received *ex vivo* αβ^+^ T cell- and CD19^+^ B-cell-depleted peripheral blood stem cells (PBSCs). Prior to 2012, haploHSCT was performed using CD3/CD19^−^ depleted or CD3^−^depleted grafts; only three patients in this cohort underwent transplantation during this earlier period. Donors received granulocyte colony-stimulating factor (G-CSF) for at least 4 days before stem cell collection, which was performed on day-−1. αβ^+^ T cells were depleted using the CliniMACS® system (Miltenyi Biotec, Bergisch Gladbach, Germany) in accordance with the manufacturer's instructions.

Conditioning regimens were selected according to institutional protocols and disease type, as follows:

For lymphoid malignancies, patients received 800 cGy of total body irradiation (TBI), rabbit anti-thymocyte globulin (rATG; Thymoglobulin®, 3 mg/kg), fludarabine (180 mg/m^2^), and cyclophosphamide (100 mg/kg).

Patients with myeloid malignancies, including acute myeloid leukemia (AML), refractory anemia with excess blasts (RAEB), and juvenile myelomonocytic leukemia (JMML), received TBI 600 cGy, rATG (3 mg/kg), fludarabine (180 mg/m^2^), and cyclophosphamide (100 mg/kg). For bone marrow failure syndromes, such as severe aplastic anemia (SAA) and hypoplastic myelodysplastic syndrome (MDS), the same conditioning regimen used for myeloid malignancies was applied. In patients with hemophagocytic lymphohistiocytosis (HLH), a treosulfan-based regimen (14 g/m^2^) combined with thiotepa (10 mg/kg), rATG (5 mg/kg), and fludarabine (180 mg/m^2^) was used.

### Data collection

Data were obtained through a review of electronic medical records and included: patient demographics, underlying medical conditions, reasons for PICU admission, hospital and PICU length of stay, time from haploHSCT to PICU admission, presence of acute GVHD, cytomegalovirus (CMV) reactivation, and use of inotropes, mechanical ventilator (MV), or continuous renal replacement therapy (CRRT).

To assess illness severity at PICU admission, the pediatric risk of mortality (PRISM) III, pediatric sequential organ failure assessment (pSOFA), and oncological pediatric risk of mortality (O-PRISM) scores were calculated within the first 24 h of admission. Clinical parameters required for these scores were calculated, including cardiovascular and neurological indicators (heart rate, mean arterial pressure, and Glasgow coma scale score); acid–base/blood gas values; biochemical (glucose, creatinine, potassium, blood urea nitrogen, and bilirubin), and hematological parameters (white blood cell count, platelet count, and coagulation profile).

The primary outcomes were 30-day from the time of PICU admission and in-hospital mortality. Risk factors associated with these mortality outcomes were also identified. Thirty-day mortality was selected to capture early critical illness mortality, while in-hospital mortality was chosen to reflect the complete outcomes of patients managed in the PICU setting. Longer-term endpoints such as 100-day and 30-day mortality, which are standard in transplant outcome studies, were beyond the scope of this PICU-focused analysis and will be considered in future investigations.

### Statistical analysis

Categorical variables were expressed as frequencies and percentages and compared using the chi-squared test or Fisher's exact test, as appropriate. Continuous variables were presented as mean ± standard deviation (SD) for normally distributed data or median with interquartile range (IQR) for non-normally distributed data. Between-group comparisons for continuous variables were conducted using the two-tailed Student's *t*-test.

Univariate logistic regression analyses were performed to evaluate associations between clinical variables and outcomes. A *p*-value of <0.05 was considered statistically significant. All statistical analyses were conducted using SPSS (version 21.0; IBM Corp, Armonk, NY, USA).

## Results

Among patients who underwent haploPBSCT, 78 were admitted to the PICU between 2010 and 2023. According to the exclusion criteria, four patients who died within 24 h and 11 patients aged 18 years or older were excluded. Consequently, 63 patients were included in the final analysis.

### Baseline characteristics

The median age at PICU admission was 12.55 years (IQR: 4.63–14.69), and 37.1% (*n* = 26) of the patients were male. Leukemia was the most common underlying diagnosis, and respiratory complications were the most frequent reason for ICU admission. There is no significant difference in transplant characteristics. The mean hospital length of stay was 127.68 days, while the mean PICU length of stay was 21 days. The mean interval from haploPBSCT to PICU admission was 194 days. Acute GVHD was observed in 22.9% (*n* = 16) of patients, and CMV reactivation was present in 47.1% (*n* = 33).

### Mortality and risk factors

The 30-day mortality from the time of PICU admission and in-hospital mortality rates were 33.3% and 50.8%, respectively. In *t*-test analyses, hospital length of stay, PICU length of stay, presence of chronic GVHD, and PRISM score were significantly associated with 30-day mortality (*p* = 0.026, *p* = 0.019, *p* = 0.030, *p* = 0.047, respectively; Table 1). For in-hospital mortality, significant variables identified by *t*-test analysis included the cause of PICU admission, PICU length of stay, and need for MV (*p* = 0.02, *p* = 0.043, *p* = 0.004; Table 2).

**Table 1:** Comparison of clinical outcomes between survivor and non-survivor of 30-day hospital mortality.

Variables	Survival (*N* = 42)	Non survival (*N* = 21)	*p*-value
Age, yr, median (IQR)	12.99 (5.67–14.73)	8.17 (3.66–14.47)	0.55
Male, *n* (%)	19 (45.2)	7 (33)	0.424
Body weight (kg)	36.43 ± 20.3	28.32 ± 15.47	0.084
Underlying disease			0.423
Leukemia	21 (50)	11 (52.4)	
Lymphoma	0	1 (4.8)	
Non-malignancy	18 (42.9)	7 (33.3)	
Solid tumor	2 (4.8)	2 (9.5)	
Transplant characteristics			
CD34+ cell dose (× 10^6^/kg)	7.30 ± 4.12	7.55 ± 3.99	0.834
Time to neutrophil engraftment (days)	10.6 ± 3.10	10.68 ± 1.57	0.890
Time to platelet engraftment (days)	21.16 ± 9.83	18.93 ± 8.02	0.426
Cause of ICU admission			0.084
Respiratory	20 (47.6)	12 (57.1)	
Cardiac	1 (2.4)	0	
Monitoring during chemotherapy	6 (14.3)	1 (4.8)	
Neurologic	6 (14.3)	0	
Gastrointestinal	1 (2.4)	2 (9.5)	
Kidney	6 (14.3)	2 (9.5)	
Infection	2 (4.8)	4 (19)	
Hospital length of stay (day)	143.02 ± 85.03	97 ± 69.26	*0*.*026*
ICU length of stay (day)	26.48 ± 30.5	10.14 ± 7.42	*0*.*019*
Time from haploHSCT to ICU admission (day)	166.4 ± 172.87	249.24 ± 414.27	0.389
Acute GVHD	19 (45.2)	10 (47.6)	0.757
Chronic GVHD	4 (9.5)	8 (38.1)	*0*.*030*
Skin	2	2	
Gastrointestinal	2	5	
Lung	2	2	
CMV reactivation	25 (59.5)	8 (38.1)	0.12
PRISM	11.95 ± 5.51	15.76 ± 7.5	0.047
PSOFA	7.54 ± 3.72	7.75 ± 3.74	0.841
O-PRISM	19.76 ± 8.7	21.14 ± 6.74	0.491
Use of Inotropics	13 (31)	8 (38.1)	0.584
Use of Ventilator	29 (69)	18 (85.7)	0.222
Use of CRRT	18 (42.9)	10 (47.6)	0.791

Data are presented as mean ± standard deviation for continuous variables and *n* (%) for categorical variables, unless indicated otherwise.

ICU, intensive care unit; HaploPBSCT, haploidentical peripheral blood stem cell transplantation; GVHD, graft versus host disease; CMV, cytomegalovirus; PRISM, pediatric risk of mortality; PSOFA, pediatric sequential organ failure assessment; O-PRISM, oncological pediatric risk of mortality; CRRT, continuous renal replacement therapy.

**Table 2:** Comparison of clinical outcomes between survivor and non-survivor of in-hospital mortality.

Variables	Survivor (*N* = 31)	Non survivor (*N* = 32)	*p*-value
Age, yr, median (IQR)	8.64 (4.22–14.24)	12.66 (4.67–15.56)	0.508
Male	14 (45.2)	12 (37.5)	0.613
Body weight (kg)	34.09 ± 20.61	33.38 ± 17.82	0.885
Underlying disease			0.807
Leukemia	16 (51.6)	16 (50)	
Lymphoma	0	1 (3.1)	
Non-malignancy	12 (38.7)	13 (40.6)	
Solid tumor	2 (6.5)	2 (6.2)	
Transplant characteristics			
CD34+ cell dose (x 10^6^/kg)	7.89 ± 4.19	6.80 ± 3.88	0.321
Time to neutrophil engraftment (days)	10.14 ± 0.79	11.1 ± 3.65	0.171
Time to platelet engraftment (days)	19.83 ± 8.47	21.18 ± 10.26	0.631
Cause of ICU admission			*0*.*02*
Respiratory	10 (32.3)	22 (68.8)	
Cardiac	1 (3.2)	0	
Monitoring during chemotherapy	6 (19.4)	1 (3.1)	
Neurologic	6 (19.4)	0	
Gastrointestinal	1 (3.2)	2 (6.2)	
Kidney	5 (16.1)	3 (9.4)	
Infection	2 (6.5)	4 (12.5)	
Hospital length of stay (day)	141.87 ± 93.65	113.94 ± 68.77	0.181
ICU length of stay (day)	14.23 ± 17.4	27.63 ± 31.6	0.043
Time from haploHSCT to ICU admission (day)	180.87 ± 194.33	206.78 ± 341.25	0.712
Acute GVHD	17 (54.8)	12 (37.5)	0.307
Chronic GVHD	4 (12.9)	8 (25)	0.335
Skin	2	3	
Gastrointestinal	2	5	
Lung	2	2	
CMV reactivation	18 (58.1)	15 (46.9)	0.453
PRISM	11.84 ± 6.18	14.56 ± 6.51	0.093
PSOFA	6.62 ± 3.67	8.45 ± 3.57	0.062
OPRISM	19.84 ± 8.51	20.59 ± 7.74	0.714
Use of Inotropics	10 (32.3)	11 (34.4)	1
Use of Ventilator	18 (58.1)	29 (90.6)	*0*.*004*
Duration of ventilator	12.57 ± 22	26.71 ± 34.56	0.335
Use of CRRT	11 (35.5)	17 (53.1)	0.207

Data are presented as mean ± standard deviation for continuous variables and *n* (%) for categorical variables, unless indicated otherwise.

ICU, intensive care unit; HaploPBSCT, haploidentical peripheral blood stem cell transplantation; GVHD, graft versus host disease; CMV, cytomegalovirus; PRISM, pediatric risk of mortality; PSOFA, pediatric sequential organ failure assessment; O-PRISM, oncological pediatric risk of mortality; CRRT, continuous renal replacement therapy.

To identify risk factors associated with 30-day mortality from the time of PICU admission, univariate and multivariate logistic regression analyses were performed. Chronic GVHD was identified as a significant independent risk predictor of 30-day mortality [*p* = 0.043, OR: 4.515 (95% CI: 1.050–19.414)] (Table 3). For in-hospital mortality, the same analyses demonstrated that the need for MV was an independent prognostic factor associated with a more than fivefold increase in the risk of mortality [*p* = 0.046, OR: 5.15 (95% CI: 1.029–25.724); Table 4]. Although the mean duration of MV differed by approximately 10 days between the two groups, this difference was not statistically significant.

**Table 3:** Multivariate logistic regression analysis: 30-day mortality.

Variables	Univariate logistic regression analysis		Multivariate logistic regression analysis	
	*P*-value	OR (95% CI)	*P*-value	OR 95% CI
Hospital length of stay	0.044	0.992 (0.984–1.000)		
ICU length of stay	0.040	0.949 (0.903–0.998)		
Chronic GVHD	0.011	5.846 (1.507–22.673)	0.043	4.515 (1.050–19.414)
PRISM	0.042	1.103 (1.003–1.212)		

**Table 4:** Multivariate logistic regression analysis: in-hospital mortality .

Variables	Univariate logistic regression analysis		Multivariate logistic regression analysis	
	*P*-value	OR (95% CI)	*P*-value	OR 95% CI
Causes of ICU admission	0.774	0.704 (0.064–7.742)		
ICU length of stay	0.064	1.025 (0.999–1.052)		
Use of ventilator	0.006	6.981 (1.745–27.926)	0.046	5.15 (1.029–25.724)

**Figure 2:**
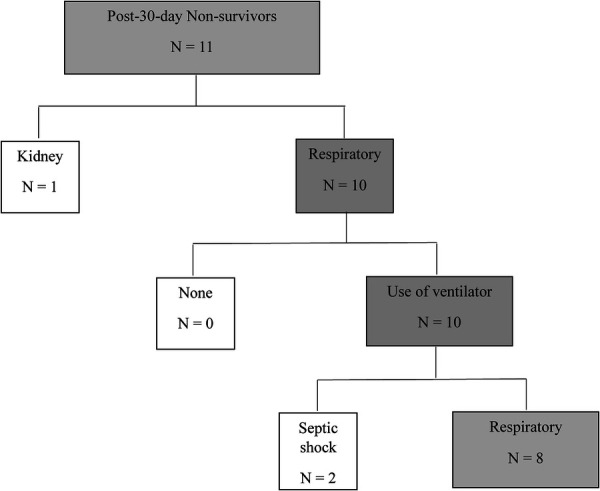
Distribution of causes of death and ventilator use in post-30-day non-survivors after haploPBSCT.

Among patients who survived beyond 30 days after PICU admission, 11 were subsequently classified as non-survivors at in-hospital mortality. Of these, 10 patients had been admitted with respiratory failure and required ventilatory support. Despite intervention, 8 of these patients died from progressive respiratory failure, most commonly due to pneumonia (Figure 2). No significant association was observed between in-hospital mortality and the duration of MV [*p* = 0.088, OR: 1.019 (95% CI: 0.997–1.042)].

## Discussion

Among patients who underwent haploPBSCT, the 30-day mortality rate after ICU admission was 33.3%, and the in-hospital mortality rate was 50.8%. Respiratory failure was the predominant cause of ICU admission and the leading cause of death in both groups. The need for MV emerged as a significant independent predictor of in-hospital mortality. Most patients who died during hospitalization had been admitted with respiratory failure and required ventilator support, with pneumonia representing the most frequent cause of death.

The clinical characteristics and post-transplant complications of *ex vivo* αβ⁺ T-cell depleted haploPBSCT differ substantially from those of conventional allogenic PBSCT. Beyond donor compatibility, these modalities diverge in graft composition and immune reconstruction dynamics. In αβ^+^ T-cell depleted haploPBSCT, selective depletion of αβ⁺ T-cells and CD19⁺ B cells reduces the risk of both acute and chronic GVHD, while preserving *γδ* T-cells and NK cells, which mediate early innate immune defense and graft-vs.-leukemia activity (16, 17). Post-transplant cyclophosphamide (PTCy)-based haploHSCT offers the advantage of broad accessibility and technical simplicity, making it one of the most widely adopted platforms for haploidentical transplantation. However, as a high-dose myeloablative agent, cyclophosphamide is known to exert significant endothelial toxicity, which has been associated with an increased incidence of endothelial dysfunction events such as veno-occlusive disease and transplant-associated thrombotic microangiopathy (18–22). In this regard, αβ^+^ T-cell depletion-based haploPBSCT may offer a potential advantage by circumventing the need for high-dose PTCy, thereby mitigating the risk of endothelial complications. Nevertheless, this approach is not without its own limitations. Although αβ^+^ depletion effectively reduces alloreactivity and the risk of GVHD, it is associated with a prolonged period of immune reconstitution, during which patients remain highly susceptible to viral, bacterial, and fungal infections (23). This vulnerability is particularly pronounced compared with PTCy-based haploHSCT, in which a broader T-cell repertoire is preserved following immune reconstitution, potentially offering greater protection against opportunistic infections (24). The predominance of infectious complications and respiratory failure observed in our cohort may, in part, reflect this inherent immunological gap specific to the αβ^+^ depletion approach particularly during the early post-transplant period (25).

Chronic GVHD necessitates prolonged systemic immunosuppression, which in turn increases infection risk (26). In this study, chronic GVHD appeared to contribute to early mortality within 30 days of ICU admission, likely through organ dysfunction. Persistent infection events and the ongoing requirement for immunomodulatory therapy to control GVHD may further exacerbate immune suppression, rendering patients increasingly susceptible to recurrent infections (26). This self-perpetuating cycle of infection and immunosuppression likely contributed to the elevated in-hospital mortality observed in this cohort.

Given the high burden of infectious complications in this population, strategies to mitigate infection-related morbidity warrant consideration. Prophylactic antifungal therapy and pre-emptive CMV monitoring with early antiviral intervention have been shown to reduce infection-related mortality in HSCT recipients (27–29). Furthermore, cellular therapies such as CD45RA-depleted donor lymphocyte infusion have demonstrated promising results in controlling viral infections in the αβ-depleted haploidentical HSCT setting, potentially interrupting the cycle of immunosuppression and recurrent infection associated with chronic GVHD (30, 31). Systematic implementation of these strategies may improve outcomes in critically ill patients following haploPBSCT.

Several studies have examined outcomes in critically ill patients following alloPBSCT. Lafarge et al. reported a 90-day overall survival rate of 51.8% among alloPBSCT recipients, identifying MV and vasopressor use as significant predictors of outcomes (15). Similarly, Gournay et al. observed an in-hospital mortality rate of 51%, again identifying MV as an independent risk factor (32). In other investigations involving both autologous and allogeneic HSCT recipients, 30-day mortality rates among patients requiring MV ranged from 55.8% to 63.2% (32).

These findings align with the results of our study, which demonstrated an in-hospital mortality rate of approximately 50% and identified MV as an independent risk factor for mortality. This underscores the poor prognosis associated with ICU admission for respiratory failure in post-transplant patients. We initially hypothesized that pulmonary manifestations of chronic GVHD might contribute to this outcome. However, our findings did not support this assumption. Instead, most ICU admissions for respiratory failure were due to infectious complications—primarily pneumonia—rather than GVHD-related pulmonary exacerbation. Pneumonia also accounted for the majority of deaths in this cohort.

In this study, MV use was significantly associated with in-hospital mortality but not 30-day mortality. This discrepancy may be explained by the observation that 10 of the 11 patients who died after surviving 30 days required MV for pneumonia-induced respiratory failure. All 11 patients received ventilator support within one month of ICU admission and remained dependent until death. Although the difference in ventilator use duration between survivors and non-survivors was not statistically significant, the mean duration differed by more than 10 days, suggesting that extended ventilator support may contribute to mortality. These findings imply that increased mortality may be more closely associated with difficulty in overcoming respiratory infections during the weaning and recovery phases than with MV duration itself.

This study has several limitations. First, it was conducted at a single center, with a relatively small sample size, limiting the statistical power and generalizability of the findings. Non-significant trends observed may reach significance in larger cohorts. Second, heterogeneity in the underlying diseases resulted in variation in chemotherapy intensity and conditioning regimens. However, as over half of the cohort had leukemia and approximately 40% had other hematologic diseases, major differences in the conditioning regimen protocols were unlikely. Moreover, as there was no significant difference in conditioning regimens between survivors and non-survivors, this suggests minimal impact on the overall outcomes. Third, changes in conditioning approaches over the study period (2010–2023), which reflected evolving clinical practice, were not fully accounted for in the analysis. The 13-year study period encompasses significant changes in transplant supportive care and conditioning practices, which may have contributed to outcome heterogeneicty within the cohort. Moreover, our institution began performing *ex vivo* αβ^+^ T-cell and CD19^+^ B-cell depleted haploPBSCT in 2012; however, this study also included patients who underwent haploidentical transplantation prior to that year using Cd3/CD19^−^ depleted or CD3^−^ depleted grafts. Therefore, not all patients in this cohort received αβ depletion, which may represents a source of heterogeneity. Nevertheless, as only three patients were transplanted before 2012, the impact of this difference on the overall findings is considered minimal. Era-based analyses were not performed due to insufficient sample size, and future multicenter studies with larger cohorts are needed to address this question.

Despite these limitations, this study provides important data on outcomes and risk factors in critically ill pediatric patients following haploPBSCT—an area of growing clinical importance as the use of haploidentical transplantation continues to expand in pediatric practice. Although the sample size was modest, our findings suggest that the outcomes and mortality risk factors in haploPBSCT recipients are broadly comparable to those reported for alloPBSCT.

In conclusion, the mortality rate among pediatric haploPBSCT patients requiring ICU care was similar to that of patients undergoing conventional allogenic PBSCT. Respiratory failure was the predominant cause of ICU admission, and MV use was identified as an independent predictor of in-hospital mortality. Future large-scale, multicenter studies are warranted to validate these results and further elucidate strategies to improve outcomes in this vulnerable population.

## Data Availability

The data supporting the conclusions of this article are available from the corresponding author upon reasonable request, in accordance with the IRB regulations of our institution.
